# Chronic Central Nervous System Graft-Versus-Host Disease to Unravel Progressive Visual Loss and Ischemic Stroke Recurrence Post-Allogeneic Hematopoietic Stem Cell Transplant: A Case Report

**DOI:** 10.3390/ijms26052289

**Published:** 2025-03-04

**Authors:** Francesco Crescenzo, Alessandra Danese, Francesco Dall’Ora, Michelangelo Turazzini

**Affiliations:** 1 Neurology Unit, Mater Salutis Hospital, AULSS 9 Scaligera, 37045 Legnago, Italy; 2 Neurology B Unit, Department of Neurosciences, Biomedicine and Movement Sciences, University of Verona, 37124 Verona, Italy

**Keywords:** stroke, angiitis, microangiopathy, optic neuropathy, graft-versus-host disease, central nervous system

## Abstract

Chronic graft-versus-host disease (cGVHD) is a prognostically negative event following hematopoietic stem cell transplant (HSCT). While cGVHD mainly affects the muscles, skin, oral mucosa, eyes, lungs, gastrointestinal tract, and liver, central nervous system (CNS) involvement remains possible and, moreover, is rare when it occurs isolated. CNS-cGVHD can manifest with a wide spectrum of CNS disorders, including cerebrovascular diseases, autoimmune demyelinating diseases, and immune-mediated encephalitis. We present a case of 65-year-old man previously treated with HSCT presenting with progressive cerebrovascular disorder and optic neuropathy without any clear alternative causal processes except for immune-mediated CNS microangiopathy in the context of possible CNS-cGVHD, along with suggestive imaging and instrumental and laboratory findings. Starting one year after HSCT for acute myeloid leukemia, when the first cerebral ischemic event occurred and was then associated with a reduction in visual acuity, an extensive diagnostic work-up had remained inconclusive over many years, leading us to the hypothesis of CNS-cGVHD and, therefore, to the start of immunosuppressive therapy. Our experience highlighted not ignoring the possibility of cGVHD as the underlying mechanism of CNS disorder, even in the absence of other systemic presentations, once more common etiologies of CNS pathological processes have been ruled out.

## 1. Introduction

Neurological disorders have been described in about 5% of patients who have undergone allogeneic hematopoietic stem cell transplant (HSCT) for hematologic malignancy, mainly at the neuromuscular junction and motor unit level [1,2,3]. They can represent neurological manifestations of chronic graft-versus-host disease (cGVHD), even though it represents a complex disregarded entity that is hard to diagnose in the absence of other “extra-neurological” clinical manifestations [4].

The involvement of the central nervous system (CNS) as the sole manifestation of cGVHD is even more poorly characterized, making its diagnosis challenging [5].

In general, it can be divided into three main categories: cerebrovascular disease, autoimmune-demyelinating disease, and immune-mediated encephalitis [1].

The current diagnostic criteria for CNS-cGVHD requires the occurrence of typical signs and symptoms affecting, for example, the skin, oral mucosa, eyes, lungs, gastrointestinal tract, liver, and no better explanation of the involvement of the CNS, along with other supportive elements (i.e., imaging, cerebrospinal fluid (CSF), pathological examination, response to immunosuppressive therapy) [1,6].

It is important to stress that the need to demonstrate the presence of “extra-neurological” involvement for diagnosing CNS-cGVHD is based on expert consensus [6], and over time, several reports on single cases or series have been published supporting the existence of “isolated” CNS-cGVHD [2,7,8]. In a recent important retrospective study on 66 patients, more than half of the patients did not meet the currently proposed criteria for CNS-cGVHD, and in any case, the treating physician estimated that CNS-GvHD was the most probable diagnosis [8].

Clinical and radiological presentation of CNS-cGVHD is heterogeneous and might implicate toxic, allo-reactive, and autoimmune mechanisms [4,5]. Among these, small white matter vascular lesions are described as common after HSCT [7], and evidence has been provided regarding exclusive CNS immune-mediated microangiopathic involvement in transplant patients as a presentation of cGVHD [9,10].

Because there is no distinctive clinical picture or supportive laboratory and radiological biomarker for isolated CNS-cGVHD, the diagnosis relies mainly on the continued accumulation of information for overall knowledge of this entity.

Therefore, we present a clinical case of a late-onset isolated CNS involvement as immune-mediated microangiopathy (i.e., CNS angiitis) involving brain parenchyma and optic nerves in a patient who underwent HSCT.

## 2. Case Presentation

**Figure 1:**
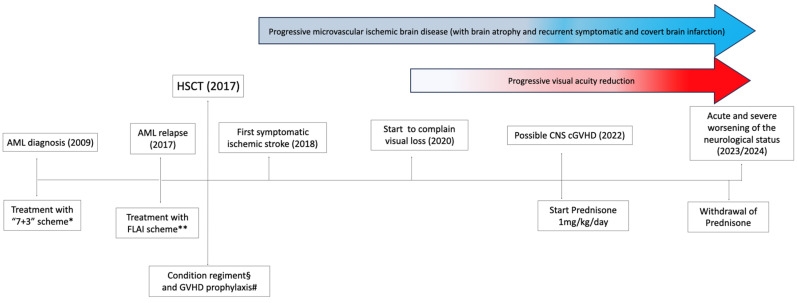
Timeline of the main patient’s clinical events and related therapy. The worsening of visual acuity and the accrual of brain lesion load are represented by arrows in red and blue, respectively.* Idarucibin and cytarabine (4 cycles of induction and consolidation); ** idarucibin, cytarabine, and fludarabine (3 cycles of induction and consolidation); § busulfan 220 mg/day for 4 days (day −6 to day −3), fludarabine 70 mg/day for 4 days (day −6 to day −3), anti-thymocyte globulin 200 and 400 mg every other day for 4 days (day −4 to day −1); # methotrexate (day +1, day +3, day +6) and cyclosporine (for 4 months).

A 65-year-old man came to our attention for progressive visual loss and signs/symptoms of cortical–subcortical microvascular ischemic brain disease. His medical history was notable for acute myeloid leukemia (AML) (French–American–British classification M4: myelomonocytic subtype) with an inversion in chromosome 16 and deletion in chromosome 7. It had manifested with signs and symptoms related to anemia and thrombocytopenia ten years earlier, was treated early with chemotherapeutic agents, and then, after a relapse occurred in 2017, was without any clinical evidence of CNS involvement, with allo-HSCT with peripheral blood stem cells from an HLA-matched sibling followed by a GVHD prophylaxis regimen including cyclosporine for four months (Figure 1). The donor was a 71-year-old female who showed cytomegalovirus (CMV) seropositivity.

**Figure 2:**
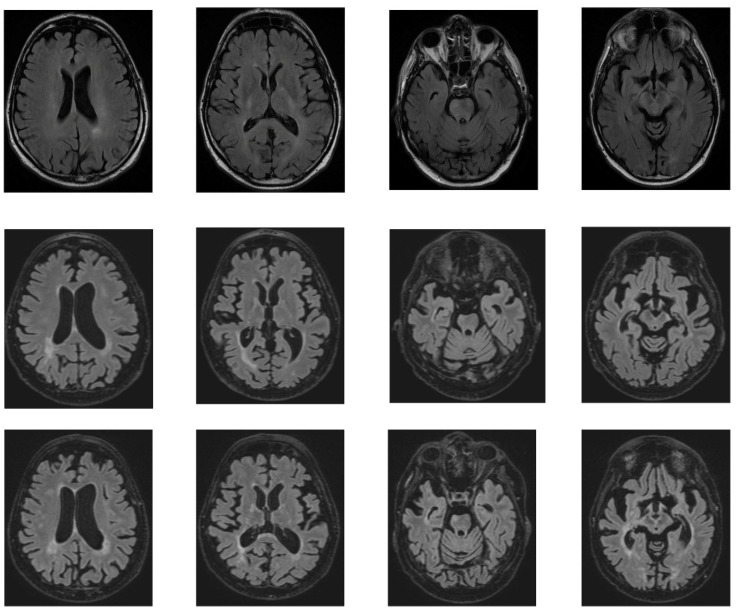
Exemplification of the evolution of brain ischemic damage over time. Axial fluid-attenuated inversion recovery (FLAIR) MRI images showing the progressive accumulation of subcortical microinfarcts associated with the development of cortical–subcortical brain atrophy. **Top** row (2018), **middle** row (2022), **bottom** row (2023).

**Figure 3:**
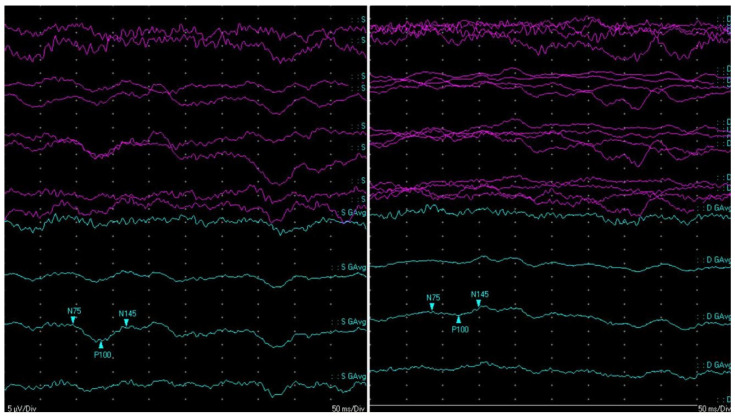
Visual conduction pathway assessment. The reversal pattern of VEP showed a predominant reduction in the amplitude of P100 in both eyes (worse on the right panel—right eye), which was not associated with a substantial modification of latency.

The first cerebral ischemic event manifested as left hemiparesis that occurred one year later (in 2018). No recognizable etiology of the ischemic stroke was found, and an insertable cardiac monitoring device was implanted. Starting two years after this first cerebral event, the patient slowly developed a progressive decrease in visual acuity and severe physical and cognitive deterioration. He underwent multiple brain magnetic resonance images (MRIs), which showed an accumulation of (sub)acute lacunar brain infarction, i.e., small (<20 mm in diameter) ischemic lesions that affect the CNS white and grey matter, as well as diffuse brain and optic nerve atrophy (Figure 2). Several serological screening tests for common infections, rheumatologic/systemic vasculitis, and inherited or acquired coagulation disorders were negative over time, except for an isolated lupus anticoagulant (LAC) positivity, which was not confirmed after at least 12 weeks. Carotid and transcranial doppler ultrasounds, MR angiography, and cardiac electronic devices–remote interrogations were steadily unremarkable. During the hospitalization at our clinic in 2022, CSF analysis showed an increased level of protein (71.3 mg/dL-n.v.: 15.0–45.0 mg/dL) and intrathecal oligoclonal bands (OCBs), together with its analogous presence in the peripheral blood (pattern type 4—“mirror”). Neither leukocytes nor red blood cells nor increased lactate levels were present in the CSF. Testing for antibodies for paraneoplastic syndrome was negative. At the same time, the CSF multiplex–polymerase chain reaction assay ruled out neuroinfectious diseases, and CNS intravascular neoplasms appeared unlikely since radiological evaluation, CSF cytopathology and flow cytofluorimetry were both negative, and the disease course was not rapidly progressive. Visually evoked potentials (VEPs) showed bilateral abnormalities, which were worse on the right side, mainly due to axonal damage (Figure 3), and a fundoscopic inspection did not demonstrate pathologic findings. Optical coherence tomography and fundus fluorescein angiography were not performed. No strategic infarct at the visual cortex was evident on brain MRI. Nerve conduction studies showed normal conduction velocities, latency, and amplitudes in the limbs’ main motor and sensory nerves; a needle electromyography was within limits.

**Figure 4:**
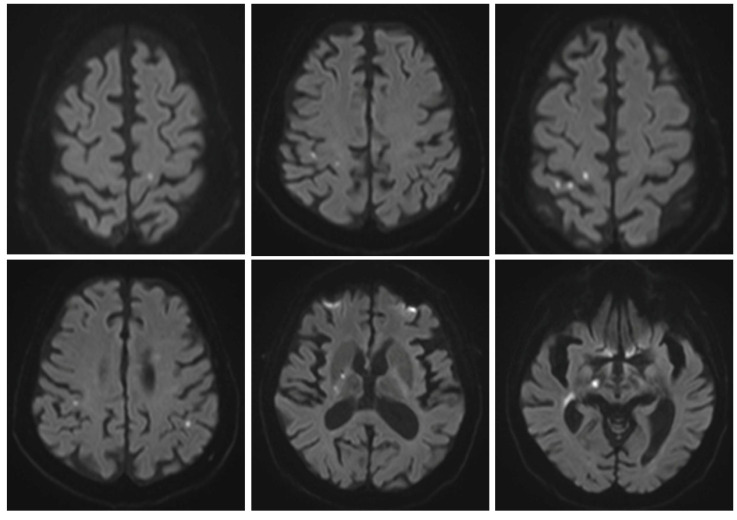
Cerebral acute microangiopathy. Axial MRI diffusion-weighted images (DWI) showing multiple bilateral small acute infarcts that occurred after steroid withdrawal.

Based on these data, ischemic immune-mediated cerebral and optic nerve complications of HSCT as possible manifestations of cGVHD were suspected. Therefore, the patient was placed on immunosuppressive treatment with steroids (1 mg/kg/day prednisone). Unfortunately, after almost a year of treatment without worsening of the neurological status, the steroid dosage was slowly tapered over eight weeks due to the occurrence of adverse effects (mood swings, fluid retention, elevated blood pressure, dermatitis, and ecchymosis), with a subsequent flare-up of neurological symptoms (dysphagia, dysarthria, worsening of left hemiparesis, challenges with visual perception, confusion, and disorientation in familiar places) and MRI evidence of new cerebral acute ischemic foci, causing a worsening of the patient’s neurological status (Figure 4) three months after stopping the steroid treatment. At the end of 2023, after a reprised laboratory-instrumental diagnostic work-up, including a second CSF analysis confirming only an increased level of protein (84 mg/dL) in the absence of pleocytosis (0, 000 10*3/uL leukocytes), the patient’s care target was directed to comfort measures until his death a year later. Remarkably, during the overall follow-up, the patient showed no cutaneous and visceral manifestations of cGVHD or evidence of systemic thrombus formation. Post-transplant immune-mediated thrombotic microangiopathy/angiitis involving brain parenchyma and optic nerves could better explain the clinical picture as a conceivable presentation of isolated CNS involvement of cGVHD.

## 3. Discussion

Our case highlights the possibility of isolated CNS involvement as a manifestation of cGVHD, defined as a CNS involvement that cannot be better explained in patients who have undergone HSCT, even in the absence of “extra-neurological” involvement of cGVHD. This diagnosis is supported by excluding alternative diagnoses, such as classic etiologies of ischemic stroke, infections, neurotoxicity, or other known CNS demyelinating disorders.

A diagnosis of CNS-cGVHD remains difficult because of its nonspecific presentation, although it is important because of its association with an unfavorable prognosis [2,8].

Our clinical case does not fulfill either Openshaw’s criteria of CNS-cGVHD (2009) [6] or Grauer’s proposed definition of “possible” CNS-cGVHD (2010) [1]. The only criterion missing for diagnosing (possible) CNS-cGVHD is systemic involvement typical of cGVHD. In this regard, several case series of CNS-cGVHD have been described even without “extra-neurological” involvement [9,10,11]. Our case could also align with neuropathological reports indicating that inflammatory CNS vasculopathies (i.e., angiitis) result from GVHD [9,10,12,13]. Even if the definitive diagnosis is biopsy-proven, CSF abnormalities and neuroradiological findings, although heterogeneous, according to affection vessels of different sizes, could help identify this subtle condition in these patients. In transplanted patients, Sakellari et al. reported cerebrovascular accidents, distinguished in thrombotic microangiopathy (TMA), thromboembolic events, and CNS hemorrhage, were observed throughout the whole period of follow-up, either in the early post-transplant period (<100 days—mainly TMAs) or in the late post-transplant period (>100 days—both TMAs and thromboembolic events) [14]. For these complications, no consistent preventive therapies are known.

Response to immunosuppressive agents (i.e., steroids and other agents, such as cyclophosphamide) is deemed a criterion for CNS-cGVHD according to Openshaw’s criteria [6]. However, it is considered unnecessary for diagnosis according to Grauer’s proposed criteria [1], since a lack of response to immunosuppressive agents could happen in patients with CNS-cGVHD [14]. In the case series published by Ruggiu et al., 70% of patients reported partial response to corticosteroid treatment, and about 20% reported treatment failure [10].

Bone marrow transplantation is noted to be a risk factor for cerebrovascular accidents [15], but, in our case, what argues against this conclusion is the bilateral optic neuropathy, which, in our opinion, was probably due to the immune-mediated microangiopathic process affecting small arteries that provide blood supply to optic nerves. In addition, Wang et al. reported a case of GVHD-induced optic neuropathy after allogeneic HSCT [16].

In our case, a limitation in the field of investigation for visual pathway disturbances is that the results of optical coherence tomography and fundus angiography are unavailable. These techniques, which study ocular disorders with high sensitivity, provide valuable information on the severity and progression of the disease [17].

A possible alternative immune mechanism underlying cerebrovascular involvement could be suspected by LAC positivity. However, the role of its isolated positivity, despite being associated with major vascular events [18], remains primarily debated, and to assume a causal role of thrombotic events requires a second confirmatory positivity, which did not occur in our case. In this regard, previous studies showed a poor predictive value for a thrombotic event for isolated LAC, since it is often observed in elderly patients in the absence of clinical symptoms or on a first occasion and is not then remotely confirmed. In addition, it has been demonstrated that LAC positivity can be due to the interference of other autoimmune antibodies [19]. These laboratory data, together with the presence of OCBs in the serum and CSF, lead us to interpret it as a long-lasting immune-mediated neurovascular phenomenon post-allogenic HSCT. The worsening and recurrence of cerebrovascular events during steroidal tapering are aligned with this hypothesis.

In this setting, the relationship between donor and recipient CMV serostatus and HSCT outcomes is also worthy of note. There are studies showing that CMV seronegativity is associated with lower mortality [20]. Some other studies have reported that both donor and recipient seropositivity favor the HSCT outcome only in transplantation with an unrelated donor [21]. A seminal study conducted by Jacobsen et al. has shown that donor CMV seropositivity predisposes to cGVHD as a result of immunity reactivation of donor cells against recipient’s CMV-infected cells [22].

In terms of differential diagnostic work-up, it is worth mentioning that the patient was treated with cyclosporine after transplantation. This drug is known to be related to CNS toxicity, presenting most often with posterior reversible encephalopathy syndrome and other leukoencephalopathies [23,24]. However, these complications tend to be present as an acute encephalopathy with diffuse white matter involvement and usually reverse with stopping drug treatment [7,24]. In contrast, our patient started to exhibit the first neurological symptoms long after cyclosporine had already been withdrawn. Furthermore, primary drug-induced damage to the CNS was not suspected, since the patient had never previously undergone intrathecal administration of chemotherapy. Furthermore, CNS involvement in AML occurs typically as leptomeningeal infiltration or parenchymal mass lesions, which were both lacking in our case, and this occurs more frequently in pediatric patients [25,26]. AML CNS involvement remains a cause of mortality in a very short period [27], while instead, our patient slowly accumulated, over time, a severe physical disability with paresis, parkinsonism, pseudobulbar signs, and prevalent cognitive impairment related to the recurrence of ischemic strokes beyond a decline in visual acuity.

## 4. Conclusions

Due to the high risk of unfavorable long-term outcomes in patients who suffer CNS disorders after HSCT, it is advisable not to ignore the possibility of cGVHD as the underlying mechanism, even in the absence of classic systemic presentation, once more common etiologies have been excluded [2,10].

The need for further comprehension of the clinical course of patients with CNS-cGVHD highlights the importance of further research into identifying correct prevention strategies, such as determining risk factors. Likewise, CNS involvement could also hypothetically precede a more classic cGVHD clinical picture, figuring as a hierarchic condition of more diffuse and systemic disease potentially permitting the diagnosis of cGVHD in more patients. Further studies are needed to explore these hypotheses in clinical practice.

## Data Availability

Data is contained within the article.

## Abbreviations

AML	Acute myeloid leukemia
CNS	Central nervous system
CSF	Cerebrospinal fluid
HLA	Human Leukocyte Antigen
CMV	Cytomegalovirus
FLAIR	Fluid-attenuated inversion recovery
GVHD	Graft-versus-host disease
HSCT	Hematopoietic stem cell transplant
LAC	Lupus anticoagulant
TMA	Thrombotic microangiopathy
MRI	Magnetic resonance imaging
OCB	Oligoclonal bands
VEP	Visual evoked potentials
